# Transfer of mitochondria via tunneling nanotubes rescues apoptotic PC12 cells

**DOI:** 10.1038/cdd.2014.211

**Published:** 2015-01-09

**Authors:** X Wang, H-H Gerdes

**Affiliations:** 1 Department of Biomedicine, University of Bergen, Jonas Lies vei 91, Bergen 5009, Norway

## Abstract

Tunneling nanotubes (TNTs) are F-actin-based membrane tubes that form between cells in culture and in tissues. They mediate intercellular communication ranging from electrical signalling to the transfer of organelles. Here, we studied the role of TNTs in the interaction between apoptotic and healthy cells. We found that pheochromocytoma (PC) 12 cells treated with ultraviolet light (UV) were rescued when cocultured with untreated PC12 cells. UV-treated cells formed a different type of TNT with untreated PC12 cells, which was characterized by continuous microtubule localized inside these TNTs. The dynamic behaviour of mCherry-tagged end-binding protein 3 and the accumulation of detyrosinated tubulin in these TNTs indicate that they are regulated structures. In addition, these TNTs show different biophysical properties, for example, increased diameter allowing dye entry, prolonged lifetime and decreased membrane fluidity. Further studies demonstrated that microtubule-containing TNTs were formed by stressed cells, which had lost cytochrome *c* but did not enter into the execution phase of apoptosis characterized by caspase-3 activation. Moreover, mitochondria colocalized with microtubules in TNTs and transited along these structures from healthy to stressed cells. Importantly, impaired formation of TNTs and untreated cells carrying defective mitochondria were unable to rescue UV-treated cells in the coculture. We conclude that TNT-mediated transfer of functional mitochondria reverse stressed cells in the early stages of apoptosis. This provides new insights into the survival mechanisms of damaged cells in a multicellular context.

Apoptosis is an important regulatory mechanism of tissue homeostasis. It is triggered by the extrinsic pathway through the activation of proapoptotic receptors or by the intrinsic pathway through the destabilization of mitochondria in response to various forms of cell injury or stress.^1^ Notably, stressed cells are also strongly influenced by intercellular communicative networks. This includes diffusible growth factors, cytokines and other small molecules secreted from neighbouring cells, which can modulate the fate of distressed cells. For example, stem cells release growth factors to protect dysfunctional neurons in the brain.^2^ In tumour stroma, activated fibroblasts are thought to promote tumour progression by secreting growth factors that act in a paracrine manner.^3^ Moreover, contact-dependent signalling, for example, via adhesion molecules, can trigger contact inhibition or protection of endothelial cells.^4^ In addition, gap junctions have been shown to be involved in the transfer of death or survival molecules in different cell types.^5^ Therefore, the signals transferred from neighbouring cells influence the viability of target cells through different pathways.

In 2004, our group described a previously unrecognized form of cell-to-cell interaction based on nanoscaled, F-actin-containing membrane tubes.^6, 7^ These tubes, referred to as membrane or tunneling nanotubes (TNTs), were subsequently found in numerous cell types in culture and in tissues.^8, 9, 10, 11^ Importantly, TNTs facilitate the intercellular exchange of diverse cellular signals and components ranging from electrical signalling to organelles.^12, 13, 14, 15^ Moreover, pathogens such as human immunodeficiency virus (HIV) and prions can spread between cells along TNTs.^16, 17^ Consistent with the model that TNTs are involved in cell-to-cell communication, apoptosis regulators may be transferred via TNTs between apoptotic and healthy cells to alter the fate of recipient cells. Indeed, it has been shown that TNTs can propagate the death signal Fas ligand between T lymphocytes to induce cell death.^18, 19^ TNTs have been also proposed to participate in the rescue of injured cardiomyoblasts or endothelial cells by mesenchymal stem cells (MSCs) through transferred mitochondria.^20 ,21^ However, the rescue mechanism by how and when this event was accomplished remains elusive.

In this study, we found that PC12 cells stressed by ultraviolet (UV) radiation were rescued from apoptosis when cocultured with untreated, healthy PC12 cells. Single-cell analysis showed that stressed cells in the early stages of apoptosis form a new type of TNT to interact with untreated cells. These TNTs have a distinct cytoskeletal composition and biophysical properties when compared with TNTs interconnecting normal PC12 cells. We also observed the presence and transport of mitochondria in the TNTs formed by stressed cells. Notably, the rescue effect was inhibited when the formation of TNTs were impaired by incubating with an F-actin-depolymerizing drug, or when the mitochondria of rescuer cells were damaged. Our results suggest that the delivery of functional mitochondria via TNTs mediates the recovery of PC12 cells in the early stages of apoptosis.

## Results

### TNTs abate stress-induced apoptosis of PC12 cells

**Figure 1:**
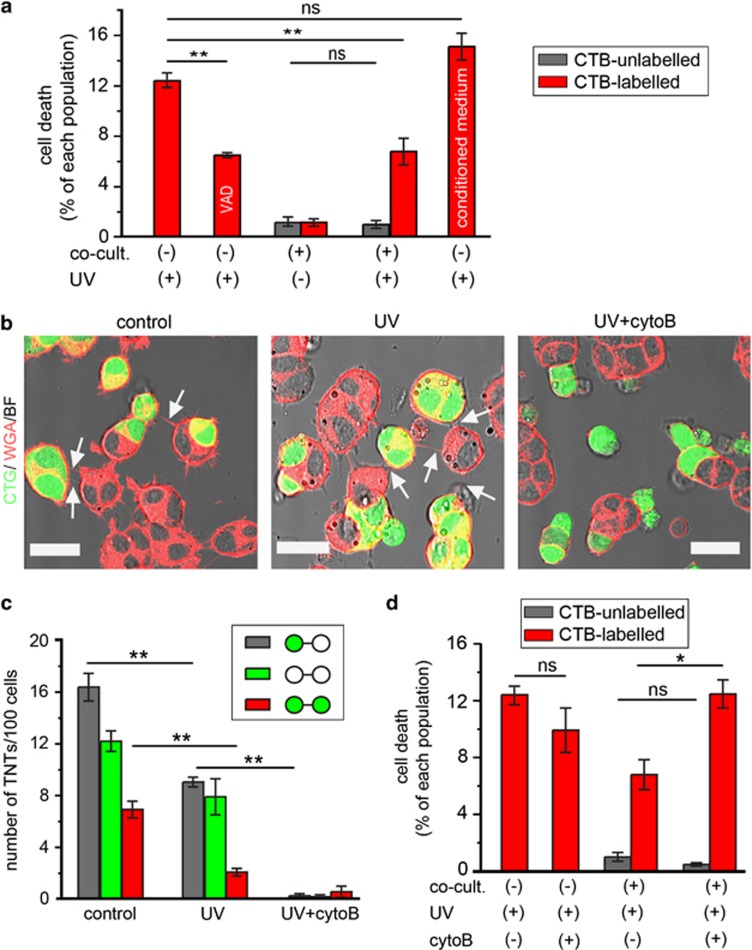
Rescue of UV-treated PC12 cells in cocultures. (**a**) The level of cell death of UV-treated cells decreased in coculture condition. CTB-labelled PC12 cells were treated with UV light and grown in monoculture or in coculture with untreated cells for 24 h. The percentage of dead cells was quantified by staining with annexin V-AF488. UV-treated cells were also incubated for 24 h in the presence of 80 *μ*M Z-VAD-FMK or in conditioned medium from untreated cells as indicated. (**b**) TNTs (arrows) were formed between CTG-labelled (CTG, green) and -unlabelled cells in control and UV treatment conditions but not in the presence of 350 nM cytoB (UV+cytoB). The cocultures were stained with WGA-AF594 (WGA, red) and imaged by confocal microscope. Scale bars, 20 *μ*m. (**c**) Three hundred and fifty nanomolar cytoB abolished the formation of TNTs. The number of TNT between CTG-labelled cells (green circles), CTG-unlabelled cells (white circles) or CTG-labelled and -unlabelled cells were counted in the conditions as in (**b**). (**d**) Three hundred and fifty nanomolar cytoB inhibited the rescue effect. CTB-labelled cells treated with UV were cocultured with untreated cells (unlabelled) in the absence or presence of 350 nM cytoB for 24 h. The percentage of dead cells in both cell populations was quantified. NS, not significant; *P<0.05; **P<0.01

PC12 cells form numerous TNTs that can facilitate intercellular transfer of vesicles and membrane-associated proteins.^6, 22^ To investigate whether TNTs have a role in the transfer of apoptosis regulators, we first established a coculture system of apoptosis-induced and healthy PC12 cells. Apoptosis of cells labelled with CellTracker Blue (CTB) was induced by UV treatment, which could be attenuated by the pancaspase inhibitor Z-VAD-FMK (*P*=0.005, one-way ANOVA test with Dunnett's *post hoc* test; Figure 1a). Next, UV-treated or -untreated cells labelled with CTB were cultured either alone or together with untreated cells at a 1 : 1 ratio. The quantification of dead cells revealed no increase of cell death in the untreated population 24 h after the beginning of the cocultures (*P*=0.7402, *t*-test; Figure 1a, grey bars). However, there were significantly less dead cells of the UV-treated population in the coculture compared with the monoculture condition (*P*=0.006, one-way ANOVA test with Dunnett's *post hoc* test; Figure 1a, red bars). This suggests that the UV-treated cells were rescued. This rescue effect did not occur when UV-treated cells were incubated with conditioned medium collected from untreated cells (*P*=0.341, one-way ANOVA test with Dunnett's *post hoc* test; Figure 1a), indicating a contact-dependent mechanism.

Therefore, we investigated TNT connectivity in the coculture by staining the plasma membrane with wheat germ agglutinin (WGA) conjugated to Alexa Fluor-594 (Figure 1b). Approximately 10 TNTs per 100 cells were formed between CellTracker Green (CTG)-labelled cells treated with UV and untreated cells, albeit fewer than under control condition (*P*=0.0007, one-way ANOVA test with Tukey's *post hoc* test; Figure 1c, grey bars). In agreement with our previous study on acute myeloid leukaemia cells,^23^ TNT formation in PC12 cells was impaired after UV treatment (*P*=0.0001, one-way ANOVA test with Tukey's *post hoc* test; Figure 1c, red bars) and under other apoptosis stimulations (valinomycin, cisplatin or actinomycin D), resulting in increased cell death, which correlated with a decrease in TNT formation (Supplementary Figures 1a and b, red bars). This decrease was partly reversed by the Z-VAD-FMK (Supplementary Figure 1b, grey bars), suggesting that the inhibitory effect on TNT formation is caspase-dependent. Moreover, CTG-stained cells contained less TNTs than unstained cells in control condition (Figure 1c). This is because the PC12 cells are prone to form clusters with time, which reduce the chance of TNT formation (data not shown).

To test whether TNTs have a role in the rescue of stressed cells, the cocultures were treated with 350 nM cytochalasin B (cytoB), which is an F-actin-depolymerizing agent shown to abolish TNT formation.^22^ Consistently, cytoB treatment inhibited the formation of TNTs between CTG-labelled cells irradiated with UV and untreated cells (Figure 1b, right; Figure 1c, grey bars, *n*=4, *P*<0.0001, one-way ANOVA test with Tukey's *post hoc* test). Three hundred and fifty nanomolar cytoB alone did not increase cell death in monocultures of UV-treated cells (*P*=0.1561, *t*-test) or in untreated cells in coculture (*P*=0.1819, *t*-test; Figure 1d). However, the percentage of dead cells in the UV-treated population in coculture was significantly higher in the presence than in the absence of cytoB (*P*=0.017, *t*-test; Figure 1d). To confirm if the rescue effect is general in PC12 cells, CTB-labelled cells were treated with 50 *μ*M actinomycin D (ac-D) for 2 h to induce apoptosis and then cocultured with untreated cells for 24 h. We observed a similar rescue effect (*P*=0.0012, *t*-test; Supplementary Figure 2), which could be inhibited by cytoB (*P*=0.0003, *t*-test). These data clearly demonstrate that cytoB prevents the rescue of cell death observed in cocultures and suggest that TNTs may be involved in the rescue process.

### Structural changes of TNTs formed by stressed PC12 cells

**Figure 2:**
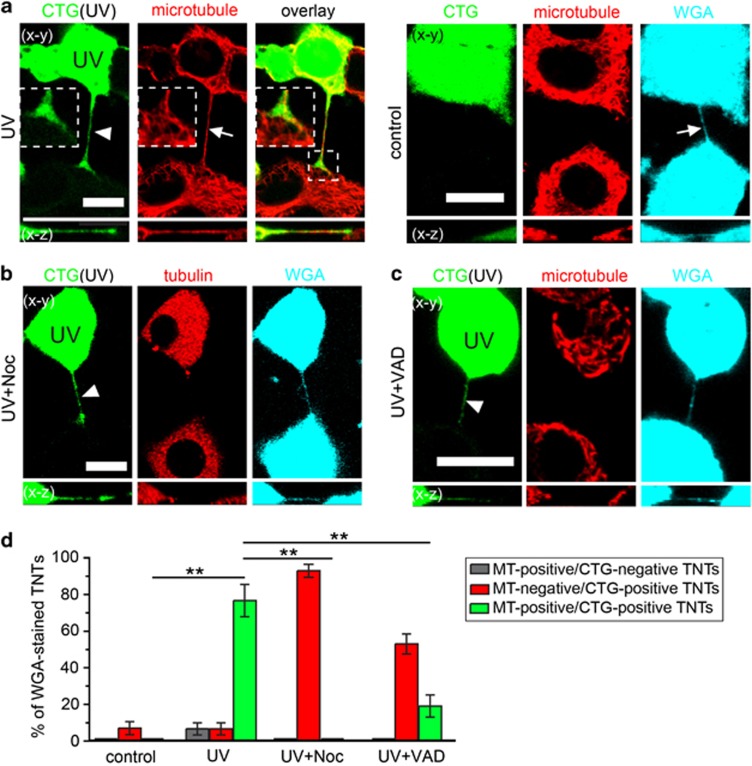
UV-treated PC12 cells form TNTs containing microtubules. (**a**) TNT formed by UV-treated cells (CTG-labelled, green) contained CTG (arrowhead) and microtubules (arrow). (Right) TNTs (arrow) formed by control cells did not contain CTG and microtubules. The cells were stained with WGA-AF633 (WGA, cyan) and immunostained with anti-*α*-tubulin antibody to visualize microtubules (red) and imaged by confocal microscope. (**b** and **c**) When the cocultures were treated with 10 *μ*M Noc or 80 *μ*M Z-VAD-FMK for 24 h, the CTG-TNTs (arrowheads) did not contain microtubules. (**d**) Statistical evaluation of frequencies of marker occurrence in TNTs between CTG-labelled and -unlabelled cells in the conditions as in (**a**–**c**). The ratio of TNTs containing microtubules but no CTG (grey bars), CTG but no microtubules (red bars) or microtubules and CTG (green bars) to total WGA-stained TNTs were calculated. Scale bars, 10 *μ*m. **P<0.01

To understand the role of TNTs in the rescue process, we performed in-depth microscopy analysis of TNTs connecting UV-treated and -untreated cells. CTG-labelled cells were exposed to UV light and subsequently cocultured with untreated cells. Confocal imaging showed that the CTG dye diffused into the TNTs of UV-treated cells but did not pass into the connected untreated cells (Figure 2a, left). By contrast, CTG was absent from TNTs connecting control cells (Figure 2a, right), which is consistent with our previous finding that a dye with low molecular weight does not enter TNTs formed between normal PC12 cells.^6^ The entry of CTG into this special type of TNTs may be because of the larger diameter of TNTs of UV-treated cells compared with TNTs of control cells (*P*=0.0483, *t*-test; Supplementary Figure 3a).

It has been reported that thicker TNTs may contain microtubules.^24^ We discovered that microtubules localized in CTG-positive TNTs (CTG-TNTs) by immunostaining of *α*-tubulin (Figure 2a, left). Further analysis revealed that 76.7±8.8% of immunostained TNTs were positive for both CTG and microtubules (*n*=30; Figure 2d, green bar). Only 6.7±3.3% of microtubule-containing TNTs (MT-TNTs) displayed no CTG staining (*n*=30; Figure 2d, grey bar), indicating that CTG-TNTs are the main population of MT-TNTs. When microtubules were completely disrupted by 10 *μ*M nocodazole (Noc), there were still CTG-TNTs despite the depolymerized microtubules (tubulin; Figure 2b). Moreover, CTG-TNTs were also observed when Noc was added at the time of coculture initiation (*n*=30). These data rule out an essential role for microtubules in the formation of CTG-TNTs. In some cases, depolymerized microtubules exist in CTG-TNTs in the presence of Noc (Supplementary Figures 3c and d). CTG-TNTs lacking microtubules were also found when cocultured cells were treated with 80 *μ*M Z-VAD-FMK (Figures 2c and d). This result suggests that caspases may affect the microtubule dynamics in CTG-TNTs. Despite these differences in cytoskeletal composition, the length of all the different types of TNTs was similar (*n*=30; Supplementary Figure 3b).

**Figure 3:**
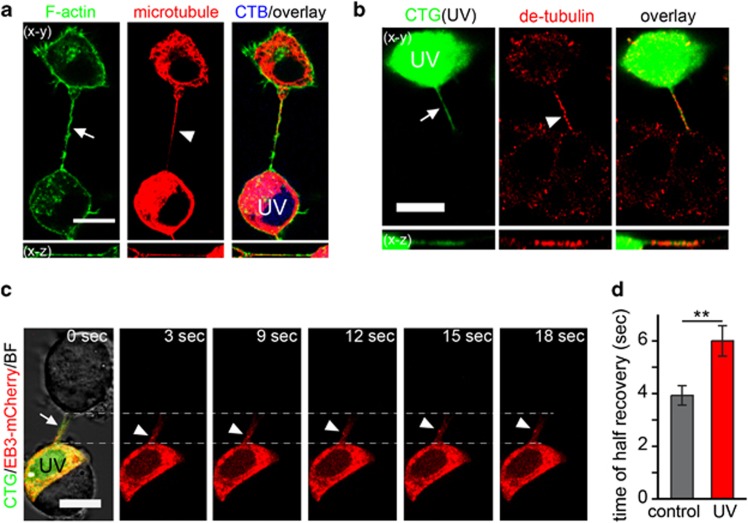
The characteristics of MT-TNTs. (**a**) The colocalization of microtubules (arrowhead) and F-actin (arrow) in a TNT between UV-treated (CTB-labelled, blue) and untreated cells. F-actin was stained with phalloidin-AF488. Microtubule was immunostained with anti-*α*-tubulin antibody and imaged by confocal microscope. (**b**) CTG-TNT (arrow) contained detyrosinated *α*-tubulin (arrowhead). CTG-labelled cells (CTG, green) were treated with UV and cocultured with untreated cells for 24 h. Cells were then immunostained with anti-detyrosinated *α*-tubulin (red) and imaged by confocal microscope. (**c**) EB3-mCherry (arrowheads) moved towards the end of a CTG-TNT (arrow). EB3-mCherry-transfected cells were labelled with CTG, treated with UV and cocultured with untreated cells for 24 h. Cells were subjected to time-lapse confocal imaging. (**d**) The membrane fluidity of CTG-TNTs was less than normal TNTs. The half-time of recovery (*t*_1/2_) of fluorescence of WGA-AF594 of TNTs between control cells (control, *n*=15) and CTG-TNTs between UV-treated and -untreated cells (UV, *n*=13) was measured by FRAP. Scale bars, 10 *μ*m. **P<0.01

Similar to the TNTs of normal PC12 cells,^6^ MT-TNTs also contained F-actin (Figure 3a). Interestingly, some microtubules were surrounded by a helix of F-actin in right-hand rotation towards the tip of TNTs (Supplementary Movie 1) with an average repeat distance of 4.1±0.3 *μ*m (*n*=5). Time-lapse imaging (90 min) revealed that 17 of 20 observed CTG-TNTs persisted, which is much longer than that of normal TNTs with a median lifetime of 7 min.^22^ This is probably due to microtubule-dependent stabilization of TNTs. In this line of thought, we found that 74.4±5.5% of CTG-TNTs contained detyrosinated tubulin (*n*=20; Figure 3b), which has been shown to increase the stability of microtubules.^25^ To evaluate the dynamics of microtubules in TNTs, we focused on the end-binding protein 3 (EB3), a protein that binds to the plus end of microtubules only during the polymerization of tubulin.^26^ Live-cell imaging demonstrated that EB3-mCherry moved towards the distal end of CTG-TNT (Figure 3c and Supplementary Movie 2) with an average velocity of 0.22±0.03 *μ*m/s (3 movies). This magnitude is comparable to the velocity of movement of EB3-GFP in neurons (0.2 *μ*m/s).^27^ Moreover, EB3-mCherry was absent from CTG-TNTs when CTG-labelled cells treated with UV were cocultured with non-treated cells expressing EB3-mCherry (*n*=16; Supplementary Figure S3e). It indicates that microtubules polymerize within CTG-TNTs from the cell body of the UV-treated cell towards the distal end of TNTs.

Besides the cytoskeleton, we investigated the membrane fluidity of CTG-TNTs by using fluorescence recovery after photobleaching (FRAP) (Supplementary Figure 3f). This analysis revealed that fluorescence recovery in CTG-TNTs was significantly longer than in TNTs of control cells (*P*=0.006, Mann–Whitney test; Figure 3d), indicating a decrease of membrane fluidity in the TNTs of the UV-treated cells. One possible reason for this decrease might be the oxidation of unsaturated phospholipids through the release of reactive oxygen species from damaged mitochondria of UV-treated cells.^28, 29^ Taken together, our data show that UV treatment of cells induces the formation of a new type of TNT with distinct cytoskeletal composition and biophysical properties.

### PC12 cells in the early stages of apoptosis form MT-TNTs

**Figure 4:**
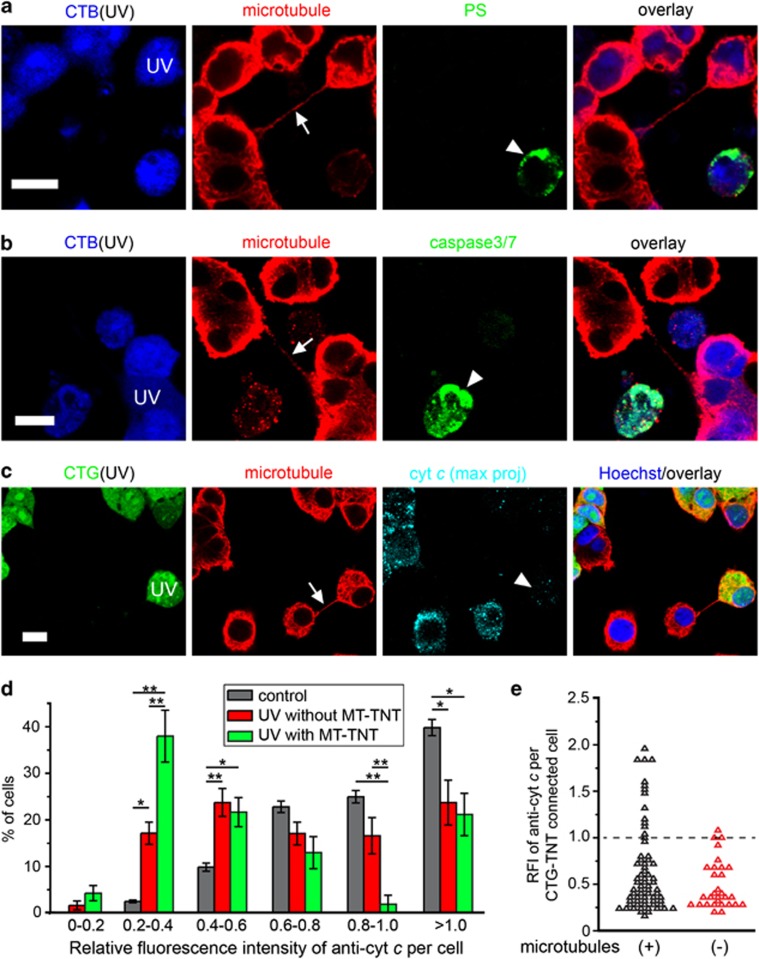
Cells in the early stages of apoptosis form MT-TNTs. (**a** and **b**) MT-TNT- (arrows) connected UV-treated cells (UV) showed neither PS exposure (**a**) nor caspase-3 activity (**b**). CTB-labelled cells (CTB, blue) were treated with UV and then cocultured with untreated cells for 24 h. The cells were stained with annexin V-AF488 (PS, green) or the Image-iT LIVE Green Caspase-3/7 Detection Kit (Caspase-3/7, green), immunostained with anti-*α*-tubulin antibody (red) and imaged by confocal microscope. Note that dead cells (arrowheads) showed PS exposure (**a**) and caspase-3/7 activity (**b**) as positive control. (**c**) MT-TNT- (arrow) connected UV-treated cell (UV) contained less cyt *c* (arrowhead) than untreated cells. CTG-labelled cells (CTG, green) were treated with UV and then cocultured with untreated cells for 24 h. The cells were immunostained with anti-*α*-tubulin (red) and anti-cytochrome *c* (cyt *c*, cyan), and imaged by confocal microscope. (**d**) UV-treated cells formed MT-TNTs when cells lose cyt *c*. The RFI of anti-cyt *c* in untreated control cells (*n*=276), UV-treated cells without MT-TNT (*n*=202) and UV-treated cells connected with MT-TNT (*n*=67) was calculated as described in the Materials and Methods section. (**e**) The frequency distribution of RFI values of anti-cyt *c* in microtubule-containing and -deficient CTG-TNT- connected UV-treated cells. CTG-labelled cells were treated with UV and then cocultured with untreated cells in the absence or presence of 80 *μ*M Z-VAD-FMK for 24 h. UV-treated cells connected with microtubule-containing CTG-TNT (*n*=67) or connected with microtubule-deficient CTG-TNT (Z-VAD-FMK treated, *n*=30) were imaged by confocal microscope and analysed as in (**d**). Scale bars, 10 *μ*m. *P<0.05; **P<0.01

To explore the formation mechanism of MT-TNTs, we investigated their appearance at different stages of apoptosis. The exposure of phosphatidylserine (PS) to the extracellular space is a landmark of apoptosis. Therefore, we analysed PS exposure in MT-TNT-connected cells by staining with annexin V coupled to AF488 (annexin V-AF488). We observed that 96.5±1.8% of MT-TNT-connected UV-treated cells were annexin V-negative (*n*=62; Figure 4a). This indicates that MT-TNTs were formed by stressed cells that did not expose PS. Another important landmark of the execution stage of apoptosis is the activation of caspase-3, which takes place earlier than the exposure of PS. However, none of the cells connected by MT-TNTs showed activation of caspase-3 (*n*=30; Figure 4b). Hence, the activation of caspase-3 is not required for the formation of MT-TNTs.

We next assessed the level of cytochrome *c* (cyt *c*) in MT-TNT-connected cells because the release of cyt *c* precedes the activation of caspase-3^30^ and UV radiation activates the intrinsic pathway of apoptosis via mitochondria destabilization. By using immunolabelling with anti-cyt *c*, we found that UV-treated cells connected with MT-TNTs displayed considerably fewer fluorescence signals of anti-cyt *c* than untreated cells (Figure 4c). To further analyse the relation between MT-TNTs and the changes of cyt *c*, we quantified the relative fluorescence intensity (RFI) of anti-cyt *c* of three groups of cells in the coculture: UV-treated cells with MT-TNTs, UV-treated cells without MT-TNTs and untreated control cells. In all, 38±5.6% of UV-treated cells with MT-TNTs displayed low RFI values, between 0.2 and 0.4, which was ~2-fold the percentage of UV-treated cells without MT-TNTs (*P*=0.006; Figure 4d). Thus, MT-TNTs were formed by UV-treated cells that released measurable amounts of cyt *c* from mitochondria but had not entered into the execution stage of apoptosis.

We previously observed that UV-treated cells labelled with CTG were able to form TNTs that contained CTG, but lacked microtubules when cocultured in the presence of the caspase inhibitor Z-VAD-FMK (Figure 2d). By using this strategy, we investigated if the MT-TNTs affect the level of cyt *c* in UV-treated cells. We found more cells with RFI values of cyt *c* above 1.0 when they were connected by CTG-TNTs containing microtubules than without microtubules (13 out of 68 and 1 out of 30 cells, respectively; Figure 4e). This suggests that the presence of microtubules in CTG-TNTs has a positive effect on the condition of UV-treated cells.

### Mitochondria are transferred and provide a rescue effect to stressed PC12 cells

**Figure 5:**
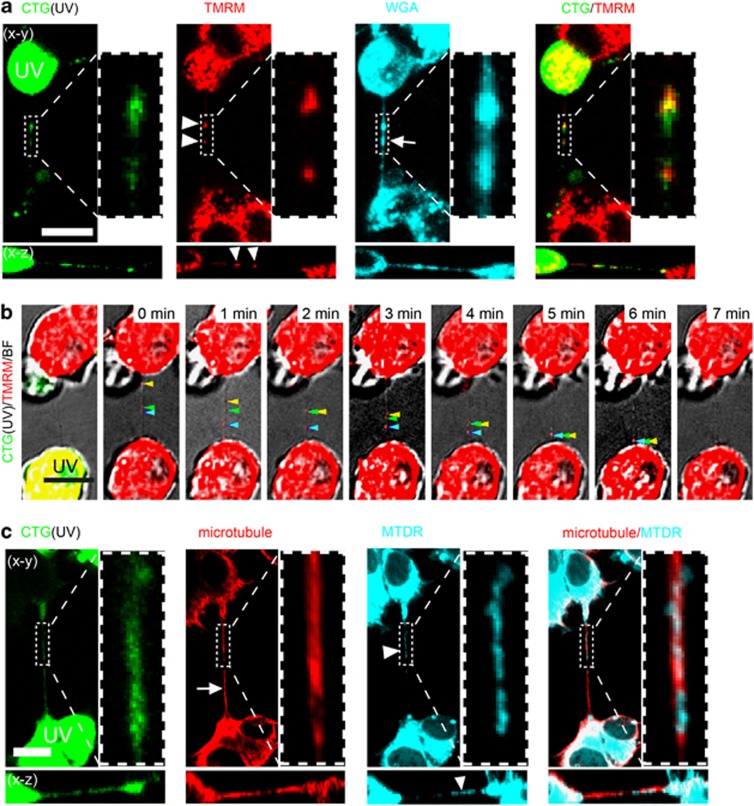
The presence and transport of mitochondria in CTG-TNTs. (**a**) Intact mitochondria (arrowheads) localized in a CTG-TNT (arrow). CTG-labelled cells (CTG, green) were treated with UV, cocultured with untreated cells for 18 h. Cells were stained with TMRM (red) and WGA-AF633 (WGA, cyan) and imaged by confocal microscope. (**b**) Mitochondria (arrowheads) translocated along a TNT towards the UV-treated cell. CTG-labelled cells (green) were treated with UV and cocultured with untreated cells for 18 h. Then, cells were stained with TMRM (red) and subjected to time-lapse confocal imaging. (**c**) Mitochondria (arrowhead) colocalized with microtubules (arrow) along a CTG-TNT. CTG-labelled cells (CTG, green) were treated with UV and cocultured with untreated cells for 24 h. The coculture was subsequently stained with MTDR (cyan), then immunostained with an anti-*α*-tubulin antibody (red) and imaged by confocal microscope. Scale bars, 10 *μ*m

The mitochondrion is a key regulator of apoptosis,^1, 31^ and its intercellular transfer has been associated with the recovery of injured cells.^20, 21, 32^ Therefore, we examined whether mitochondria are present in the TNTs derived from UV-treated cells by labelling mitochondria with the specific fluorescent dye TMRM (tetramethylrhodamine, methyl ester).^33^ We observed that TMRM-labelled mitochondria were present in CTG-TNTs connecting UV-treated and -untreated cells (Figure 5a). By contrast, TMRM-labelled mitochondria were not detected in TNTs of control cultures (data not shown). By means of live-cell imaging, we could observe TMRM-labelled mitochondria moving independently from a healthy to a UV-treated cell along the TNT (Figure 5b). The mitochondria did not migrate at uniform speeds and met together before entering the UV-treated cell (Supplementary movie 3). Unlike the velocities measured for axonal transport of mitochondria (100–1400 nm/s),^34^ the maximum speed reached by mitochondria trafficking along TNTs was ~80 nm/s (Supplementary Figure 4a). The underlying reason for this may be the smaller diameter of the TNTs, possibly imposing a higher resistance to the movement of mitochondria compared with their movement in axons. To verify the presence of mitochondria in MT-TNTs, we labelled mitochondria with MitoTracker Deep Red (MTDR) and then immunostained the microtubules. This revealed colocalization of mitochondria and microtubules in CTG-TNTs (Figure 5c), suggesting that microtubules may help the transport of mitochondria along MT-TNTs.

**Figure 6:**
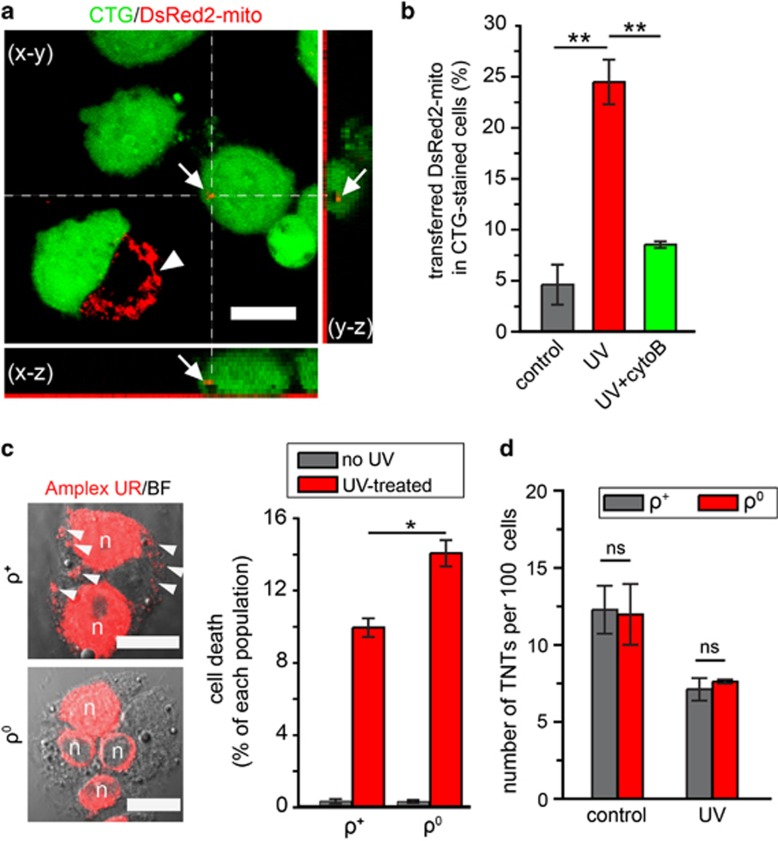
The rescue effect depends on the transfer of functional mitochondria. (**a**) The presence of DsRed2-mito (arrows) of healthy cells in a UV-treated cell. CTG-labelled cells (green) were treated with UV, cocultured with untreated cells expressing DsRed2-mito (arrowhead) for 24 h and imaged by confocal microscope. (**b**) The transfer of DsRed2-mito from healthy cells to UV-treated cells was inhibited by cytoB. The percentage of CTG-labelled cells that contained DsRed2-mito was calculated in control (without UV treatment) and in UV treatment in the absence or presence of 350 nM cytoB. (**c**) The *ρ*^0^ cells were less able to rescue the UV-treated cells than the normal cells (*ρ*^+^). Unlabelled *ρ*^0^ cells were cocultured with CTB-labelled UV-treated cells for 24 h. (Left) The *ρ*^0^ cells contained no mtDNA, whereas the *ρ*^+^ cells did (arrowheads). The *ρ*^+^ and *ρ*^0^ cells were stained with the Click-iT EdU Microplate Assay kit (Amplex UR, red) to detect EdU-labelled DNA and imaged by confocal microscope. Note that the nuclei (n) were strongly stained in both *ρ*^+^ and *ρ*^0^ cells. (**d**) The *ρ*^0^ and *ρ*^+^ cells formed comparable numbers of TNTs. TNTs formed in *ρ*^0^ and *ρ*^+^ cells were quantified under conditions with or without UV treatment (control). Scale bars, 10 *μ*m. NS, not significant; *P<0.05; **P<0.01

We next cocultured UV-treated cells with untreated cells expressing DsRed2-mito, a fluorescent mitochondrial probe, to quantify the transfer of mitochondria. 3D confocal imaging showed punctate signal of DsRed2-mito in the UV-treated cell (Figure 6a). The number of UV-treated cells containing DsRed2-mito was fivefold compared with control (*P*=0.0004, one-way ANOVA test with Tukey's *post hoc* test; Figure 6b). Notably, elimination of TNTs with 350 nM cytoB significantly decreased the transfer of DsRed2-mito (*P*=0.0016, one-way ANOVA test with Tukey's *post hoc* test; Figure 6b). Because 350 nM cytoB has no effect on endocytosis and phagocytosis of PC12 cells,^22^ our findings demonstrate that TNTs have a major role in the transfer of DsRed2-mito. Moreover, the optimum distance for the transfer of DsRed2-mito between recipient cells and closest donor cells was 9–16 *μ*m (Supplementary Figure 4b, red bars), which corresponds to the length of MT-TNTs (Supplementary Figure 3b). In contrast, there was no optimal distance for the transfer under control conditions, reflecting a random uptake of cell debris (Supplementary Figure 4b, grey bars). To obtain more evidence for the transfer of intact mitochondria, the mitochondrial DNA (mtDNA) of untreated cells was selectively labelled with the nucleoside analogue 5-ethynyl-2′-deoxyuridine (EdU). The mtDNA of transferred mitochondria was detected in UV-treated cells of the coculture (Supplementary Figure 4c). In agreement with this, it has been shown that tumour cells can acquire mtDNA from the host cells.^35^

To obtain direct evidence of a rescue effect elicited by mitochondria transferred from untreated cells, we generated PC12 cells lacking mtDNA and thus carried mitochondria deficient for functional electron transport (*ρ*^0^ cells).^36^ We observed more dead cells when UV-treated cells were cocultured with *ρ*^0^ cells than with cells carrying functional mitochondria (*ρ*^+^ cells) (*P*=0.0099, *t*-test; Figure 6c). Importantly, the number of TNTs formed by *ρ*^+^ and *ρ*^0^ cells was similar under both control and UV conditions (*P*=0.9099 and *P*=0.5339 respectively, *t*-test; Figure 6d). Taken all together, our data suggest that stressed cells form MT-TNTs and obtain transferred mitochondria that participate in the rescue effect.

## Discussion

The formation of TNTs by externally stressed cells has been addressed in several studies.^37, 38, 39^ However, those studies only considered changes in the quantity of TNTs during apoptosis. Here, we discovered that stressed cells form a new type of TNTs with different biochemical and biophysical characters. Moreover, the active movement of EB3 and the accumulation of detyrosinated tubulin in these TNTs indicate that they are highly regulated structures as opposed to cellular protrusion passively pulled by the connected cells. Importantly, our study revealed that only cells containing damaged mitochondria at a very early apoptotic stages, before the activation of caspase-3, form MT-TNTs. This suggests that cells at the execution phase of apoptosis do not form this novel type of TNT and may not be rescued by healthy cells. Interestingly, we observed that the formation of MT-TNTs did not depend on the activation of caspase-3. However the pancaspase inhibitor disturbed the entry or assembly of microtubules within TNTs. Therefore, other cysteine proteases may be involved in the formation of MT-TNTs.

The presence of mitochondria in TNTs has been shown for various cell types in different conditions, for example, between human endothelial progenitor cells and rat cardiomyocytes,^40^ between MSCs and cardiomyocytes^20, 41, 42^ and between endothelial cells and cancer cells.^43^ Here, we demonstrated that mitochondria were colocalized with microtubules inside TNTs. It has been reported that short-range movement of mitochondria may occur via the actin cytoskeleton, whereas the long distance transport is microtubule-dependent.^34^ Importantly, a recent study showed that Miro1, a Rho-GTPase that helps mitochondrial movement along microtubules, regulates the transfer of mitochondria from MSCs to epithelial cells.^44^ Thus, together with the observation of the increased diameter and prolonged lifetime of MT-TNTs, our data demonstrate that this new type of TNTs possess structural determinants that support the long distance transport of organelles.

Intercellular exchange of mitochondria has been proposed as a general principle to rescue damaged cells. A previous report showed that microinjection of intact mitochondria into oocytes reduced their high susceptibility to undergo apoptosis.^45^ Spees *et al.*^46^ found that active transfer of mitochondria from adult mammalian stem cells to somatic cells rescued aerobic respiration in cells with non-functional mitochondria. Cho *et al.*^47^ reported that MSCs can transfer mitochondria to mtDNA-deficient cells and restore their mitochondrial function. Also, a recent study discovered that bone marrow-derived stromal cells protect against acute lung injury by transferring mitochondria to alveolar epithelium cells.^32^ In this line of thought, we here demonstrate that the elimination of TNTs blocked the intercellular transfer of mitochondria and inhibited the rescue effect. In addition, the inability of *ρ*^0^ cells to rescue UV-treated cells indicates that functional mitochondria are necessary. Owing to the lack of protective histones and introns, and insufficient DNA repair capacity, mtDNA is more sensitive than nuclear DNA to damage mediated by agents such as UV or ROS.^48, 49^ Therefore, taking into account that normal and mitochondria with mutant DNA can still fuse,^50^ functional mitochondria transferred by TNTs may provide complementary effects on the damaged mitochondria of the UV-stressed cells. In addition, it may also bring antiapoptosis molecules such as Bcl-2 to prevent the process of apoptosis in the recipient cells.

Overall, we provide evidence for a link between the formation of MT-TNTs, the transfer of mitochondria and the changes of rescue effect in apoptotic stressed cells. We propose a new recovery mechanism for injured cells that proliferate slowly or cannot regenerate, such as neurons and cardiomyocytes, which are prone to apoptosis after ischaemic attacks.^51^ On the other hand, it may help drug-sensitive cancer cells to acquire survival signals from drug-insensitive cells and escape death during cancer treatment. Therefore, TNT-dependent rescue effects may act as a double-edged sword in different pathological conditions and become a potential therapeutic target in the treatment of disease.

## Materials and Methods

### Cell culture and transfection

PC12 cells (rat pheochromocytoma cells, clone 251) were cultured as described.^6^ To obtain cells lacking mtDNA (*ρ*^0^ cells), PC12 cells with a low passage number were cultured for 1 month in medium containing 0.1 *μ*g/ml ethidium bromide and 50 *μ*g/ml uridine (Sigma-Aldrich Corp., St. Louis, MO, USA).^36^ During this period, cells were passaged once a week. PC12 cells were transiently transfected with the expression vector pDsRed2-mito (Clontech Laboratories, Palo Alto, CA, USA) by using Lipofectamine 2000 transfection reagent (Invitrogen, Carlsbad, CA, USA) or pEB3-mCherry (EUROSCARF, Frankfurt, Germany) by using electroporation.

### Induction of apoptosis and coculture

PC12 cells (6 × 10^4^ cells/well) were placed in *μ*-Slide 8-well chambers (Ibidi GmbH, Martinsried, Germany) coated with poly-l-lysine (0.1 mg/ml; Sigma-Aldrich) and cultured overnight. Then, cells were stained with 20 *μ*M CTB CMAC (Invitrogen) or 5 *μ*M CTG CMFDA (Invitrogen) at 37 °C for 45 min and then returned into the fresh medium. To induce apoptosis, CellTracker-labelled cells were exposed to a germicidal lamp emitting 254 nm UV-C light (TUV G15T8, Philips, Eindhoven, Netherland) for 90 s. For drug treatment, CellTracker-labelled cells were treated with 50 *μ*M ac-D (Sigma-Aldrich) for 2 h, washed three times with medium containing 1% DMSO and then cultured in the standard medium. After apoptosis-inducing treatment, CellTracker-labelled cells were cocultured with untreated and unlabelled PC12 cells (6 × 10^4^ cells/well) for 24 h. In some experiments, fresh medium supplemented with 350 nM cytoB (Sigma-Aldrich), 80 *μ*M Z-VAD-FMK (Sigma-Aldrich) or 10 *μ*M Noc (Sigma-Aldrich) was added 1 h after the start of coculture.

### Evaluation of cell death

Cells grown in various experimental conditions were stained with annexin V-AF488 (1 : 500; Invitrogen) at 37 °C for 45 min and then imaged at 400 nm (for CTB) and at 488 nm (for annexin V-AF488) with a wide-field Zeiss Axiovert 200 M fluorescence microscope (Carl Zeiss, Jena, Germany) equipped with a × 10 objective, a Polychrome V monochromator (Till Photonics, Gräfelfing, Germany), and a CCD camera (PCO AG, Kelheim, Germany) controlled by TillVision software (Till Photonics). The number of annexin V-positive cells in the CTB-labelled and -unlabelled populations was counted. Evaluation of at least 2200 cells per condition was necessary to obtain reliable estimates of cell death. The cell death index was expressed as a percentage of annexin V-positive cells in the CTB-labelled or - unlabelled population.

### Quantification of TNTs

Cocultures of CTB-labelled and -unlabelled cells were stained with wheat germ agglutinin-AF488 (WGA-AF488, 1 : 300; Invitrogen) for 5 min and subjected to live-cell imaging, as follows. Stacks of 20 images from the bottom to the top of cells were acquired at 400 nm (for CTB) and at 488 nm (for WGA-AF488) by using the Zeiss Axiovert 200 M fluorescence microscope equipped with a × 63/1.40 NA oil-immersion objective and a DAPI/FITC/TRITC filter set. For each condition, at least 20 stacks were generated within 20 min. The number of TNTs in the CTB-labelled and -unlabelled populations was counted and expressed as the number of TNTs per 100 cells.

### Immunofluorescence and membrane staining

Cocultures of CellTracker-labelled and -unlabelled cells in *μ*-Slide 8-well chambers were fixed with 4% paraformaldehyde (PFA)/4% sucrose in PBS 24 h after plating. Immunofluorescence staining was performed according to standard procedures by using the primary antibody: mouse monoclonal anti-*α*-tubulin (Sigma-Aldrich), mouse monoclonal anti-detyrosinated *α*-tubulin (Abcam, Cambridge, UK), rabbit monoclonal anti-cytochrome *c* (Abcam) and secondary antibody: goat anti-mouse-AF564 and goat anti-rabbit-AF633 (Invitrogen). WGA-AF594 or WGA-AF633 (Invitrogen) was used to stain the plasma membrane. F-actin was labelled with phalloidin-AF488 (Invitrogen). For PS staining, cells were incubated with annexin V-AF488 for 30 min before fixation. All solution contained 2 mM Ca^2+^ in the experiment of annexin V-AF488 staining. 3D confocal imaging was performed on a Leica TCS SP5 confocal microscope (Leica Microsystems GmbH, Mannheim, Germany) equipped with a resonant scanner and an HCX PL APO CS × 63/1.40 NA or a × 40/1.25 NA oil-immersion objective.

To quantify the fluorescence intensity of anti-cyt *c*, all 3D confocal imaging were performed with fixed settings in each experiment. The collection of maximum projections of 3D imaging, the selection of region of interest (ROI) and the measurement of the average fluorescence intensity of anti-cyt *c* were performed by using ImageJ (National Institutes of Health, Bethesda, MD, USA). The RFI value of anti-cyt *c* per cell was calculated by dividing the mean intensity of treated cells by the mean intensity of control cells of each experiment. The RFI value of cyt *c* in healthy cells was normalized to 1.

### FRAP analysis of membrane fluidity

Cocultures of CTG-labelled and -unlabelled cells in *μ*-Slide 8-well chambers were fluorescently stained with WGA-AF594 (1 : 300) for 10 min and then returned to the fresh medium. For control conditions without UV treatment, only TNTs that formed between CTG-labelled and -unlabelled cells were selected. For cocultures with UV-treated cells, only CTG-TNTs that formed between CTG-labelled and -unlabelled cells were selected. FRAP was carried out by using the Leica TCS SP5 confocal microscope. The ROI (3 × 10 *μ*m^2^) was centred in the middle of the TNT to ensure that a 3 *μ*m length of the TNT was bleached by the argon and HeNe laser at full power (488, 563 and 633 nm). Before bleaching, the ROI was imaged for 6 s to obtain a baseline. The ROI was then bleached for 2 s and allowed to recover for 30 s. Calculation of the half-time of recovery of fluorescence (*t*_1/2_) was performed with the Leica LAS AF Lite software (Leica Microsystems GmbH).

### Measurement of caspase-3 activity

The activity of caspase-3 was measured by using the Image-iT LIVE Green Caspase-3/7 Detection Kit (Invitrogen). Briefly, CTB-labelled (UV-treated) and -unlabelled PC12 cells were cocultured for 24 h and then incubated with FLICA solution for 90 min. Thereafter, cells were fixed with PFA and followed with immunofluorescence staining of anti-*α*-tubulin as described above. The images were acquired with the Leica TCS SP5 confocal microscope equipped with a resonant scanner and a × 40/1.25 NA oil-immersion objective with excitation wavelength of 400 nm (CTB), 488 nm (FLICA) and 561 nm (anti-tubulin).

### Imaging of mitochondria

To label mitochondria in living cells, cells were stained with 50 nM TMRM (Sigma-Aldrich) for 10 min, and then subjected to time-lapse imaging by using the Leica TCS SP5 confocal microscope equipped with a resonant scanner, a × 63/1.40 NA oil-immersion objective and a stage with 37 °C and 10% CO_2_. To detect mitochondria in fixed PC12 cells, live cells were first incubated with 1 *μ*M MTDR FM (Invitrogen) in medium at 37 °C for 40 min, returned to the fresh medium and then fixed for immunolabelling. The excitation wavelengths were 561 nm (TMRM) and 633 nm (MTDR).

To monitor the transfer of mitochondria from untreated to UV-treated cells, pDsRed2-mito transiently transfected PC12 cells were cultured for 24 h. Cells were trypsinized and cocultured with CTG-labelled and UV-treated cells for 24 h. The cells were then stained with WGA-AF633 and imaged with the Leica TCS SP5 confocal microscope equipped with a resonant scanner and a × 63/1.40 NA oil-immersion objective. ROIs (60 × 60 *μ*m^2^) that contained at least one DsRed2-mito- positive donor cell were selected for 3D imaging. For each condition, 20 ROIs with fluorescent cells and 1 ROI with only background fluorescence were selected. For analysis, a threshold value (background fluorescence) was subtracted from the 3D fluorescence images for each experiment. The number of DsRed2-mito signals (minimum 3 pixels) in CTG cells was counted in the maximum projection of the 3D images.

### Detection of mtDNA

mtDNA was detected by using the Click-iT EdU Microplate Assay Kit (Invitrogen) with modifications as described.^52^ PC12 cells were loaded with 10 *μ*M EdU for 24 h. EdU incorporates into newly synthesized nuclear DNA and mtDNA. EdU-labelled cells were cocultured with CTB-labelled and UV-treated cells for 24 h. Then, the cells were fixed and the incorporated EdU was detected with Oregon Green 488 azide through a click chemistry-based reaction. The signal of EdU-Oregon Green 488 azide complex was further amplified by incubation with anti-Oregon Green antibody conjugated to horseradish peroxidase. Horseradish peroxidase reacted with Amplex UltraRed to produce a local fluorescence signal. The imaging was performed at the Leica TCS SP5 confocal microscope equipped with a resonant scanner and a × 63/1.40 NA oil-immersion objective with an excitation wavelength of 633 nm.

### Statistical analyses

In all cases, at least three independent experiments were performed. Values are expressed as means±S.E.M. Data were analysed with either Student's two-tailed *t*-test (Microsoft Excel, Seattle, WA, USA) or one-way ANOVA test with Tukey's or Dunnett's *post hoc* test (IBM SPSS Statistics, Armonk, NY, USA). All error bars show 95% confidence intervals: *P<0.05; **P<0.01; NS, not significant.

## Abbreviations

EdU: 5-ethynyl-2′-deoxyuridine
ac-D: actinomycin D
CTB: CellTracker Blue
CTG: CellTracker Green
CTG-TNTs: CTG-positive TNTs
cytoB: cytochalasin B
cyt *c*: cytochrome *c*
EB3: end-binding protein 3
FRAP: fluorescence recovery after photobleaching
HIV: human immunodeficiency virus
MSCs: mesenchymal stem cells
MT-TNTs: microtubule-containing TNTs
mtDNA: mitochondrial DNA
MTDR: mitotracker deep red
Noc: nocodazole
PFA: paraformaldehyde
PC: pheochromocytoma
PS: phosphatidylserine
ROI: region of interest
RFI: relative fluorescence intensity
TMRM: tetramethylrhodamine, methyl ester
TNTs: tunneling nanotubes
UV: ultraviolet light
WGA: wheat germ agglutinin

## References

[bib1] 1Galluzzi L, Kepp O, Kroemer G. Mitochondria: master regulators of danger signalling. Nat Rev Mol Cell Biol 2012; 13: 780–788. 23175281 10.1038/nrm3479

[bib2] 2Martino G, Pluchino S. The therapeutic potential of neural stem cells. Nat Rev Neurosci 2006; 7: 395–406. 16760919 10.1038/nrn1908

[bib3] 3Kalluri R, Zeisberg M. Fibroblasts in cancer. Nat Rev Cancer 2006; 6: 392–401. 16572188 10.1038/nrc1877

[bib4] 4Dejana E. Endothelial cell–cell junctions: happy together. Nat Rev Mol Cell Biol 2004; 5: 261–270. 15071551 10.1038/nrm1357

[bib5] 5Decrock E, Vinken M, De Vuyst E, Krysko DV, D'Herde K, Vanhaecke T et al. Connexin-related signaling in cell death: to live or let die? Cell Death Differ 2009; 16: 524–536. 19197295 10.1038/cdd.2008.196

[bib6] 6Rustom A, Saffrich R, Markovic I, Walther P, Gerdes HH. Nanotubular highways for intercellular organelle transport. Science 2004; 303: 1007–1010. 14963329 10.1126/science.1093133

[bib7] 7Onfelt B, Nedvetzki S, Yanagi K, Davis DM. Cutting edge: membrane nanotubes connect immune cells. J Immunol 2004; 173: 1511–1513. 15265877 10.4049/jimmunol.173.3.1511

[bib8] 8Gerdes HH, Carvalho R.N. Intercellular transfer mediated by tunneling nanotubes. Curr Opin Cell Biol 2008; 20: 470–475. 18456488 10.1016/j.ceb.2008.03.005

[bib9] 9Chinnery HR, Pearlman E, McMenamin PG. Cutting edge: membrane nanotubes *in vivo*: a feature of MHC class II+ cells in the mouse cornea. J Immunol 2008; 180: 5779–5783. 18424694 10.4049/jimmunol.180.9.5779 PMC3392179

[bib10] 10Pyrgaki C, Trainor P, Hadjantonakis AK, Niswander L. Dynamic imaging of mammalian neural tube closure. Dev Biol 2010; 344: 941–947. 20558153 10.1016/j.ydbio.2010.06.010 PMC3873863

[bib11] 11Gerdes HH, Rustom A, Wang X. Tunneling nanotubes, an emerging intercellular communication route in development. Mech Dev 2013; 130: 381–387. 23246917 10.1016/j.mod.2012.11.006

[bib12] 12Wang X, Veruki ML, Bukoreshtliev NV, Hartveit E, Gerdes HH. Animal cells connected by nanotubes can be electrically coupled through interposed gap-junction channels. Proc Natl Acad Sci USA 2010; 107: 17194–17199. 20855598 10.1073/pnas.1006785107 PMC2951457

[bib13] 13Wang X, Gerdes HH. Long-distance electrical coupling via tunneling nanotubes. Biochim Biophys Acta 2012; 1818: 2082–2086. 21930113 10.1016/j.bbamem.2011.09.002

[bib14] 14Davis DM, Sowinski S. Membrane nanotubes: dynamic long-distance connections between animal cells. Nat Rev Mol Cell Biol 2008; 9: 431–436. 18431401 10.1038/nrm2399

[bib15] 15Abounit S, Zurzolo C. Wiring through tunneling nanotubes—from electrical signals to organelle transfer. J Cell Sci 2012; 125: 1089–1098. 22399801 10.1242/jcs.083279

[bib16] 16Sowinski S, Jolly C, Berninghausen O, Purbhoo MA, Chauveau A, Köhler K et al. Membrane nanotubes physically connect T cells over long distances presenting a novel route for HIV-1 transmission. Nat Cell Biol 2008; 10: 211–219. 18193035 10.1038/ncb1682

[bib17] 17Gousset K, Schiff E, Langevin C, Marijanovic Z, Caputo A, Browman DT et al. Prions hijack tunnelling nanotubes for intercellular spread. Nat Cell Biol 2009; 11: 328–336. 19198598 10.1038/ncb1841

[bib18] 18Arkwright P.D, Luchetti F, Tour J, Roberts C, Ayub R, Morales AP et al. Fas stimulation of T lymphocytes promotes rapid intercellular exchange of death signals via membrane nanotubes. Cell Res 2010; 20: 72–88. 19770844 10.1038/cr.2009.112 PMC2822704

[bib19] 19Luchetti F, Canonico B, Arcangeletti M, Guescini M, Cesarini E, Stocchi V et al. Fas signalling promotes intercellular communication in T cells. PLoS One 2012; 7: e35766. 22558220 10.1371/journal.pone.0035766 PMC3338457

[bib20] 20Cselenyak A, Pankotai E, Horvath EM, Kiss L, Lacza Z. Mesenchymal stem cells rescue cardiomyoblasts from cell death in an *in vitro* ischemia model via direct cell-to-cell connections. BMC Cell Biol 2010; 11: 29. 20406471 10.1186/1471-2121-11-29 PMC2869333

[bib21] 21Liu K, Ji K, Guo L, Wu W, Lu H, Shan P et al. Mesenchymal stem cells rescue injured endothelial cells in an *in vitro* ischemia–reperfusion model via tunneling nanotube like structure-mediated mitochondrial transfer. Microvasc Res 2014; 92: 10–18. 24486322 10.1016/j.mvr.2014.01.008

[bib22] 22Bukoreshtliev N.V, Wang X, Hodneland E, Gurke S, Barroso JF, Gerdes HH et al. Selective block of tunneling nanotube (TNT) formation inhibits intercellular organelle transfer between PC12 cells. FEBS Lett 2009; 583: 1481–1488. 19345217 10.1016/j.febslet.2009.03.065

[bib23] 23Andresen V, Wang X, Ghimire S, Omsland M, Gjertsen BT, Gerdes HH. Tunneling nanotube (TNT) formation is independent of p53 expression. Cell Death Differ 2013; 20: 1124. 23764777 10.1038/cdd.2013.61 PMC3705610

[bib24] 24Onfelt B, Nedvetzki S, Benninger RK, Purbhoo MA, Sowinski S, Hume AN et al. Structurally distinct membrane nanotubes between human macrophages support long-distance vesicular traffic or surfing of bacteria. J Immunol 2006; 177: 8476–8483. 17142745 10.4049/jimmunol.177.12.8476

[bib25] 25Janke C, Bulinski JC. Post-translational regulation of the microtubule cytoskeleton: mechanisms and functions. Nat Rev Mol Cell Biol 2011; 12: 773–786. 22086369 10.1038/nrm3227

[bib26] 26Akhmanova A, Steinmetz MO. Tracking the ends: a dynamic protein network controls the fate of microtubule tips. Nat Rev Mol Cell Biol 2008; 9: 309–322. 18322465 10.1038/nrm2369

[bib27] 27Stepanova T, Slemmer J, Hoogenraad CC, Lansbergen G, Dortland B, De Zeeuw CI et al. Visualization of microtubule growth in cultured neurons via the use of EB3-GFP (end-binding protein 3-green fluorescent protein). J Neurosci 2003; 23: 2655–2664. 12684451 10.1523/JNEUROSCI.23-07-02655.2003 PMC6742099

[bib28] 28Borst JW, Visser NV, Kouptsova O, Visser AJ. Oxidation of unsaturated phospholipids in membrane bilayer mixtures is accompanied by membrane fluidity changes. Biochim Biophys Acta 2000; 1487: 61–73. 10962288 10.1016/s1388-1981(00)00084-6

[bib29] 29Gao W, Pu Y, Luo KQ, Chang D.C. Temporal relationship between cytochrome c release and mitochondrial swelling during UV-induced apoptosis in living HeLa cells. J Cell Sci 2001; 114: 2855–2862. 11683418 10.1242/jcs.114.15.2855

[bib30] 30Goldstein JC, Waterhouse NJ, Juin P, Evan GI, Green DR. The coordinate release of cytochrome *c* during apoptosis is rapid, complete and kinetically invariant. Nat Cell Biol 2000; 2: 156–162. 10707086 10.1038/35004029

[bib31] 31Newmeyer DD, Ferguson-Miller S. Mitochondria: releasing power for life and unleashing the machineries of death. Cell 2003; 112: 481–490. 12600312 10.1016/s0092-8674(03)00116-8

[bib32] 32Islam MN, Das SR, Emin MT, Wei M, Sun L, Westphalen K et al. Mitochondrial transfer from bone-marrow-derived stromal cells to pulmonary alveoli protects against acute lung injury. Nat Med 2012; 18: 759–765. 22504485 10.1038/nm.2736 PMC3727429

[bib33] 33Scaduto Jr RC, Grotyohann LW. Measurement of mitochondrial membrane potential using fluorescent rhodamine derivatives. Biophys J 1999; 76: 469–477. 9876159 10.1016/S0006-3495(99)77214-0 PMC1302536

[bib34] 34MacAskill AF, Kittler JT. Control of mitochondrial transport and localization in neurons. Trends Cell Biol 2010; 20: 102–112. 20006503 10.1016/j.tcb.2009.11.002

[bib35] 35Rebbeck CA, Leroi AM, Burt A. Mitochondrial capture by a transmissible cancer. Science 2011; 331: 303. 21252340 10.1126/science.1197696

[bib36] 36King MP. Use of ethidium bromide to manipulate ratio of mutated and wild-type mitochondrial DNA in cultured cells. Methods Enzymol 1996; 264: 339–344. 8965707 10.1016/s0076-6879(96)64032-4

[bib37] 37Zhu D, Tan KS, Zhang X, Sun AY, Sun GY, Lee JC et al. Hydrogen peroxide alters membrane and cytoskeleton properties and increases intercellular connections in astrocytes. J Cell Sci 2005; 118: 3695–3703. 16046474 10.1242/jcs.02507

[bib38] 38Lou E, Fujisawa S, Morozov A, Barlas A, Romin Y, Dogan Y et al. Tunneling nanotubes provide a unique conduit for intercellular transfer of cellular contents in human malignant pleural mesothelioma. PLoS One 2012; 7: e33093. 22427958 10.1371/journal.pone.0033093 PMC3302868

[bib39] 39Wang Y, Cui J, Sun X, Zhang Y. Tunneling-nanotube development in astrocytes depends on p53 activation. Cell Death Differ 2011; 18: 732–742. 21113142 10.1038/cdd.2010.147 PMC3131904

[bib40] 40Koyanagi M, Brandes RP, Haendeler J, Zeiher AM, Dimmeler S. Cell-to-cell connection of endothelial progenitor cells with cardiac myocytes by nanotubes: a novel mechanism for cell fate changes? Circ Res 2005; 96: 1039–1041. 15879310 10.1161/01.RES.0000168650.23479.0c

[bib41] 41Plotnikov EY, Khryapenkova TG, Vasileva AK, Marey MV, Galkina SI, Isaev NK et al. Cell-to-cell cross-talk between mesenchymal stem cells and cardiomyocytes in co-culture. J Cell Mol Med 2008; 12: 1622–1631. 18088382 10.1111/j.1582-4934.2007.00205.x PMC3918078

[bib42] 42Ma Z, Yang H, Liu H, Xu M, Runyan RB, Eisenberg CA et al. Mesenchymal stem cell–cardiomyocyte interactions under defined contact modes on laser-patterned biochips. PLoS One 2013; 8: e56554. 23418583 10.1371/journal.pone.0056554 PMC3572044

[bib43] 43Pasquier J, Guerrouahen BS, Al Thawadi H, Ghiabi P, Maleki M, Abu-Kaoud N et al. Preferential transfer of mitochondria from endothelial to cancer cells through tunneling nanotubes modulates chemoresistance. J Transl Med 2013; 11: 94. 23574623 10.1186/1479-5876-11-94 PMC3668949

[bib44] 44Ahmad T, Mukherjee S, Pattnaik B, Kumar M, Singh S, Kumar M et al. Miro1 regulates intercellular mitochondrial transport & enhances mesenchymal stem cell rescue efficacy. EMBO J 2014; 33: 994–1010. 24431222 10.1002/embj.201386030 PMC4193933

[bib45] 45Perez GI, Acton BM, Jurisicova A, Perkins GA, White A, Brown J, Trbovich AM et al. Genetic variance modifies apoptosis susceptibility in mature oocytes via alterations in DNA repair capacity and mitochondrial ultrastructure. Cell Death Differ 2007; 14: 524–533. 17039249 10.1038/sj.cdd.4402050

[bib46] 46Spees JL, Olson SD, Whitney MJ, Prockop DJ. Mitochondrial transfer between cells can rescue aerobic respiration. Proc Natl Acad Sci USA 2006; 103: 1283–1288. 16432190 10.1073/pnas.0510511103 PMC1345715

[bib47] 47Cho YM, Kim JH, Kim M, Park SJ, Koh SH, Ahn HS et al. Mesenchymal stem cells transfer mitochondria to the cells with virtually no mitochondrial function but not with pathogenic mtDNA mutations. PLoS One 2012; 7: e32778. 22412925 10.1371/journal.pone.0032778 PMC3295770

[bib48] 48Caron F, Jacq C, Rouviere-Yaniv J. Characterization of a histone-like protein extracted from yeast mitochondria. Proc Natl Acad Sci USA 1979; 76: 4265–4269. 228293 10.1073/pnas.76.9.4265 PMC411554

[bib49] 49Chen XJ, Butow RA. The organization and inheritance of the mitochondrial genome. Nat Rev Genet 2005; 6: 815–825. 16304597 10.1038/nrg1708

[bib50] 50Youle RJ, van der Bliek AM. Mitochondrial fission, fusion, and stress. Science 2012; 337: 1062–1065. 22936770 10.1126/science.1219855 PMC4762028

[bib51] 51Mattson MP, Kroemer G. Mitochondria in cell death: novel targets for neuroprotection and cardioprotection. Trends Mol Med 2003; 9: 196–205. 12763524 10.1016/s1471-4914(03)00046-7

[bib52] 52Lentz SI, Edwards JL, Backus C, McLean LL, Haines KM, Feldman EL. Mitochondrial DNA (mtDNA) biogenesis: visualization and duel incorporation of BrdU and EdU Into newly synthesized mtDNA *in vitro*. J Histochem Cytochem 2010; 58: 207–218. 19875847 10.1369/jhc.2009.954701 PMC2803709

