# Supplementary figures and images for: Transfer of mitochondria via tunneling nanotubes rescues apoptotic PC12 cells
Source: Cell Death Differ. 2015 Jan 9;22(7):1181–91. doi: 10.1038/cdd.2014.211 (PMC4572865; doi:10.1038/cdd.2014.211)

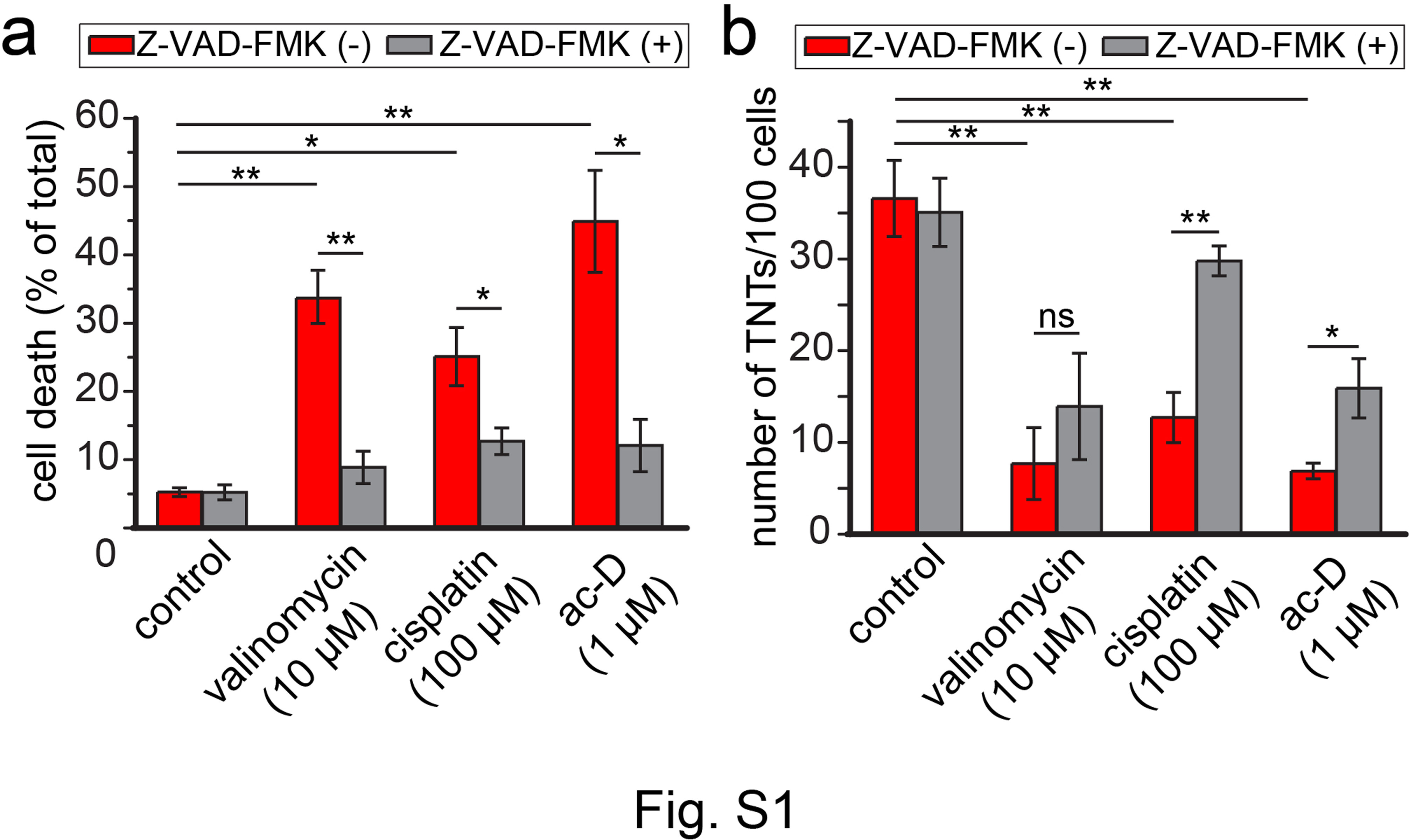

Supplement: Supplementary Figure 1 [file cdd2014211x1.tif]

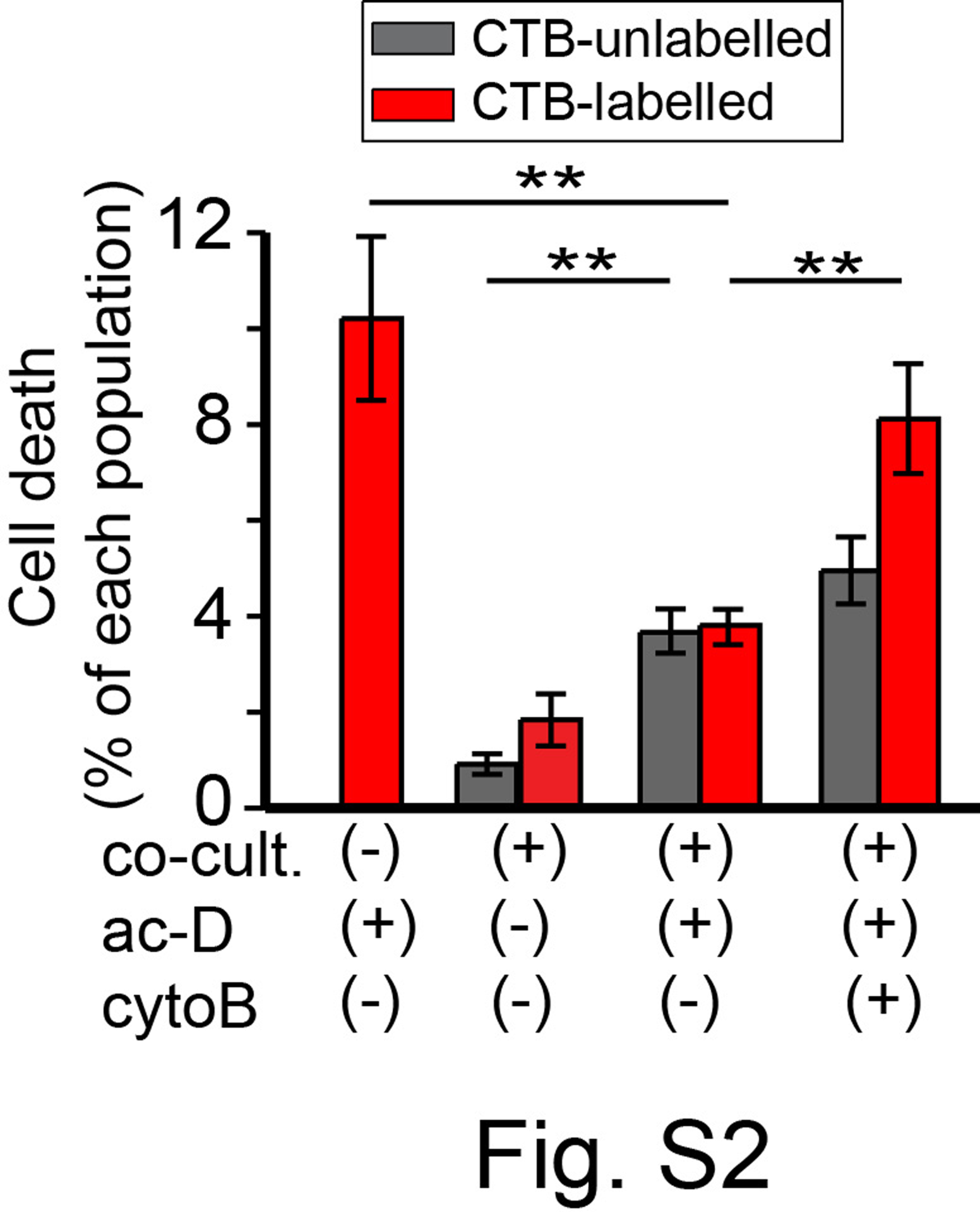

Supplement: Supplementary Figure 2 [file cdd2014211x2.tif]

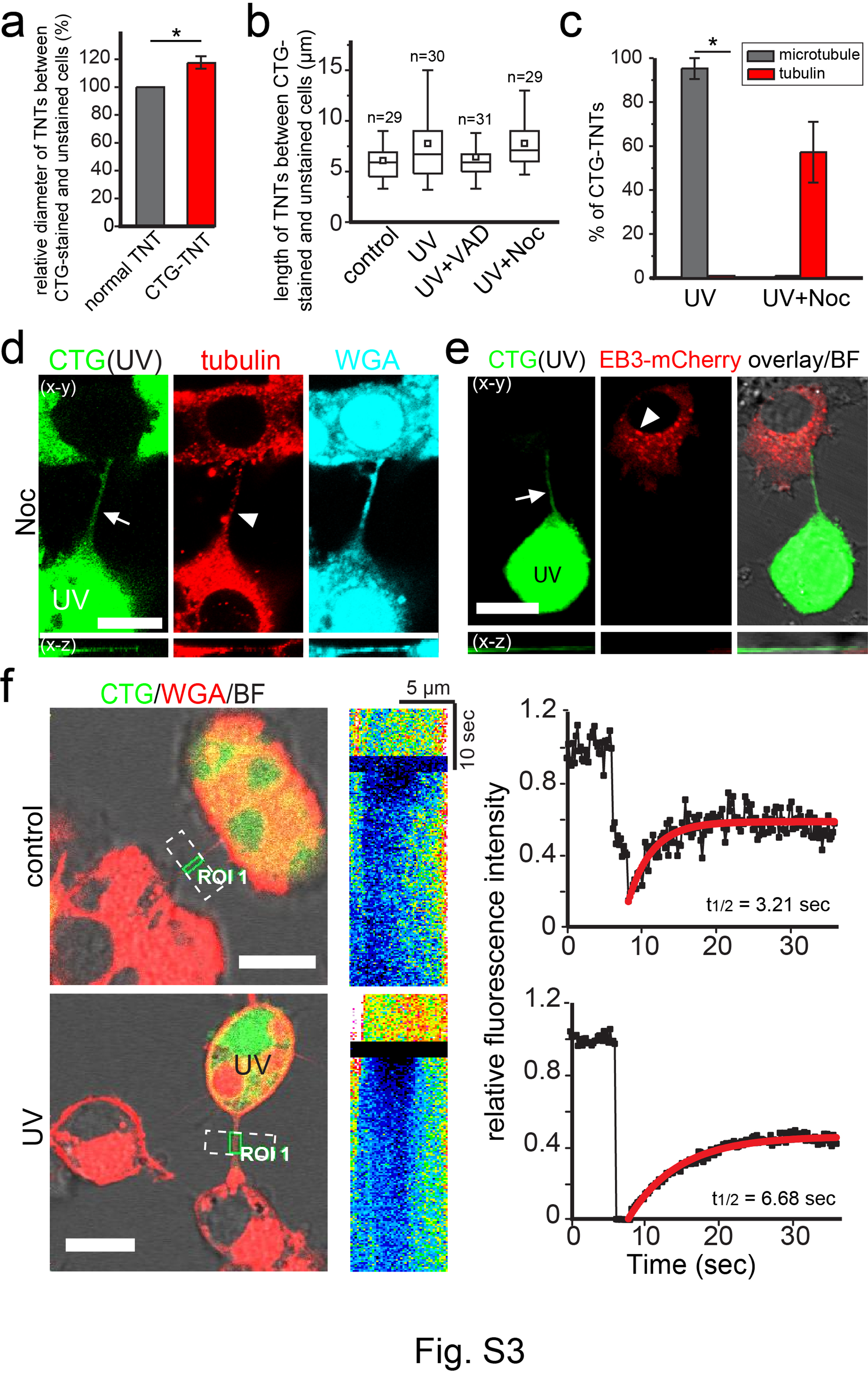

Supplement: Supplementary Figure 3 [file cdd2014211x3.tif]

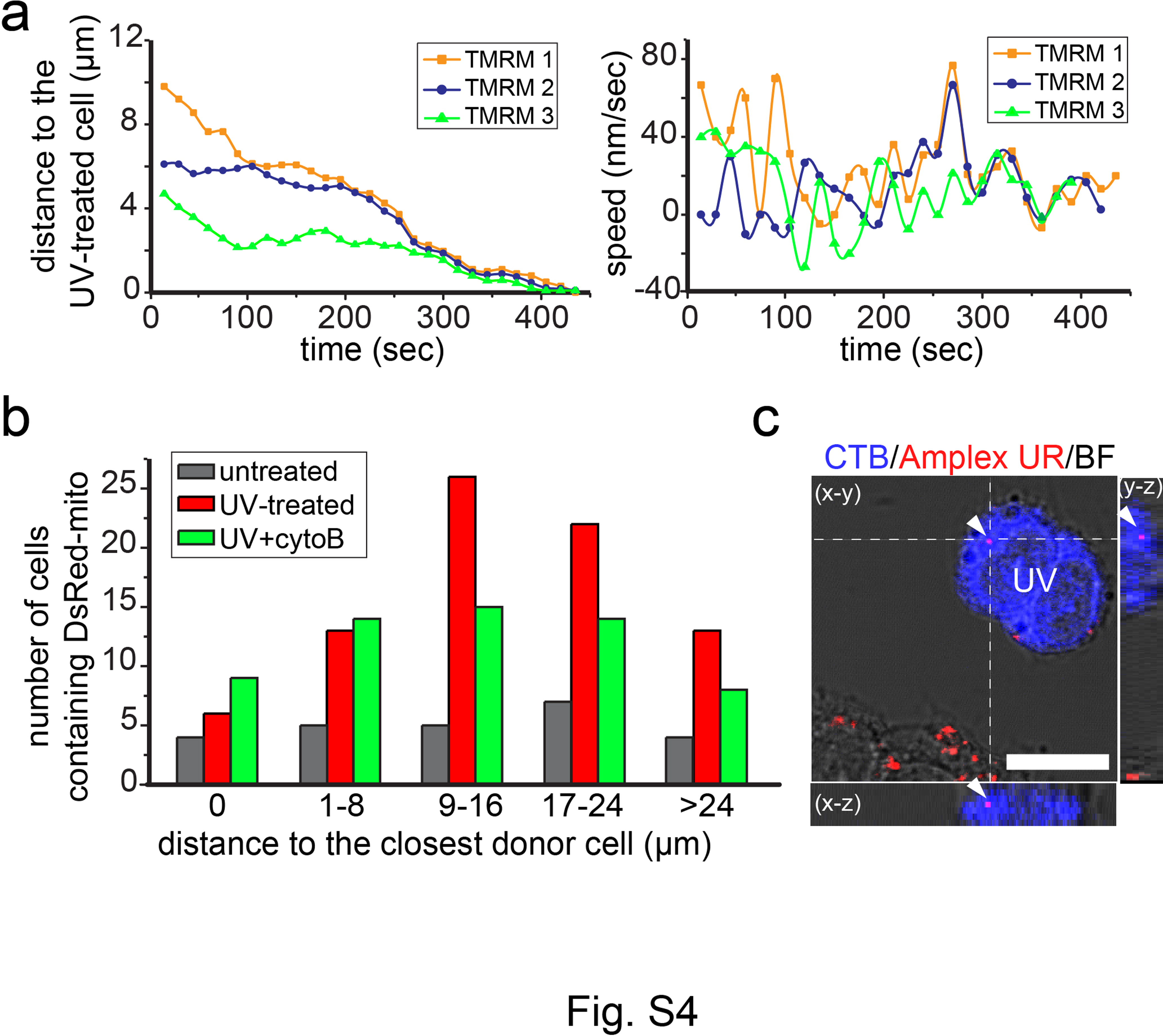

Supplement: Supplementary Figure 4 [file cdd2014211x4.tif]
