# Supplementary material for: Transfer of mitochondria via tunneling nanotubes rescues apoptotic PC12 cells
Source: Cell Death Differ. 2015 Jan 9;22(7):1181–91. doi: 10.1038/cdd.2014.211 (PMC4572865; doi:10.1038/cdd.2014.211)
Supplement: Supplementary Information [file cdd2014211x8.doc]

**Supplementary figures**

**Supplementary Figure 1** The effect of cell death on TNT formation. **(a)** PC12 cells were treated with different apoptosis inducers at the indicated concentrations for 20 hours with or without 80 μM Z-VAD-FMK. The percentage of dead cells was quantified by staining with annexin V-AF488. **(b)** The number of TNTs formed correlates inversely with the percentage of dead cells induced by each drug. Cells were treated with the indicated drugs in the presence or absence of 80 μM Z-VAD-FMK for 20 hours. The number of TNTs formed per 100 cells was quantified.

**Supplementary Figure 2** Rescue of actinomycin D-treated PC12 cells in co-cultures. CTB-labelled PC12 cells were treated with 50 μM ac-D for 2 hours and co-cultured with untreated cells in the absence or presence of 350 nM cytoB for 24 hours. The percentage of dead cells in both cell populations was quantified. Note that cell death of the untreated cells co-cultured with ac-D-treated cells was higher than in mock-treated cells (3.7 ± 0.4% versus 1.0 ± 0.2%, *n* = 6, *P* = 0.0002, *t*-test, first two grey bars). This increase was due to contamination of the culture dish with ac-D, because untreated cell cultures showed a similarly high percentage of dead cells (3.6 ± 0.3%) when grown in dishes pre-treated with ac-D.

**Supplementary Figure 3** A distinct TNT forms between UV-treated and untreated cells. **(a)** The diameter of CTG**-**TNTs was larger than normal TNTs formed in control cells. The relative diameters of middle part of TNTs were measured in 3D-confocal images of cells stained with WGA-AF633. **(b)** No difference in the lengths of CTG-TNTs formed between UV-treated and untreated cells under the conditions described in Figure 2d. Note that the TNTs formed under control condition were CTG negative. The mean value is represented by open squares and the median by horizontal lines; the 75th and 25th centiles are at the top and bottom of the boxes, respectively. **(c)** Statistical analysis of CTG-TNTs containing depolymerized microtubules in co-cultures treated with Noc. CTG-TNTs formed between UV-treated and non-treated cells in the absence or presence of 10 μM Noc (UV+Noc) for 24 hours were analysed. **(d)** CTG-TNT (*arrow*) contained depolymerized microtubules (*arrowhead*). CTG-labelled cells (CTG, *green*) were treated with UV and co-cultured with untreated cells in the presence of 10 μM Noc for 24 hours. Cells were then immunostained with anti-α-tubulin (*red*), stained with WGA-AF633 (WGA, *cyan*) and imaged by confocal microscope. (**e**) CTG-TNT (*arrow*) did not contain EB3-mCherry (*arrowhead*) from non-treated cells. CTG-labelled cells (CTG, *green*) were treated with UV, co-cultured with untreated cells expressing EB3-mCherry for 24 hours and imaged by confocal microscope. **(f)** FRAP analysis of membrane fluidity of TNTs formed between CTG-labelled and unlabelled cells. The dashed boxes in the overlay images indicate the bleached areas. The kymographs generated from the areas (*green boxes*) show the change in fluorescence intensity of WGA-AF594 over time. The half time of recovery (t_1/2_) was calculated by fitting the curves (*red*). Scale bars, 10 µm.

**Supplementary Figure 4** The transfer of mitochondria from healthy cells to UV-treated cells. **(a)** Analysis of mitochondria movement in the TNT of Figure 5b. Left: The distance between individual TMRM-labelled mitochondria (TMRM 1, TMRM 2 and TMRM 3) and the UV-treated cell over time. Note that TMRM 1 joined TMRM 2 after ~200 sec and TMRM 1 and 2 joined TMRM 3 at ~300 sec. Right: The speed of movement of three TMRM-labelled mitochondria along the TNT to the UV-treated cell. **(b)** Distance distribution between DsRed2-mito positive donor cells and acceptor cells measured from imaging data as in Figure 6b (untreated: n=25; UV-treated: n=80; UV and cytoB treated: n=60). **(c)** The presence of EdU-labelled mtDNA (*arrowheads*) of healthy cells in a UV-treated cell. CTB-labelled cells (CTB, *blue*) were treated with UV and co-cultured for 24 hours with untreated cells pre-loaded with EdU. The co-cultures were subsequently stained with the Click-iT EdU kit/Amplex UR and imaged by confocal microscope. Scale bar, 10 µm.

**Supplemental movie 1** The helix of F-actin around microtubules in a TNT. CTB-labelled cells were treated with UV and then co-cultured with untreated cells for 24 hours. F-actin was stained with phalloidin-AF488 (*green*). Microtubules were immunostained with anti-α-tubulin antibody (*red*). Images were obtained by confocal microscope. The 3D-reconstruction was generated by Imaris 3D software (Bitplane AG, Basil, Switzerland).

**Supplemental movie 2** Movement of EB3-mCherry along a TNT. EB3-mCherry transfected PC12 cells were labelled with CTG, treated with UV and co-cultured with untreated cells for 24 hours. Live confocal imaging recorded the movement of EB3-mCherry from UV-treated cell towards the tip of the TNT.

**Supplemental movie 3** Transfer of mitochondria along a TNT. CTG-labelled PC12 cells (*green*) were treated with UV and co-cultured with untreated cells for 18 hours. Subsequently, the mitochondria were stained with TMRM (*red*) and analysed by time-lapse confocal imaging. The movie shows mitochondria (*arrowheads*) moving along the TNT towards the UV-treated cell.
